# The *MC4R* genotype is associated with postpartum weight reduction and glycemic changes among women with prior gestational diabetes: longitudinal analysis

**DOI:** 10.1038/s41598-017-10101-x

**Published:** 2017-08-29

**Authors:** Aline Martins de Carvalho, Ping Shao, Huikun Liu, Han-Ling Cheng, Yan Zheng, Junhong Leng, Weiqin Li, Tao Huang, Tiange Wang, Leishen Wang, Shuang Zhang, Gang Hu, Lu Qi

**Affiliations:** 1 Department of Nutrition, University of Sao Paulo School of Public Health, Sao Paulo, Brazil; 2 Department of Nutrition, Harvard School of Public Health, Boston, Massachusetts USA; 3 Tianjin Women’s and Children’s Health Center, Tianjin, China; 4 Pennington Biomedical Research Center, Baton Rouge, Louisiana USA; 5 Department of Epidemiology, School of Public Health and Tropical Medicine, Tulane University, New Orleans, LA USA

## Abstract

The genetic variants near the Melanocortin-4 receptor gene (*MC4R*), a key protein regulating energy balance and adiposity, have been related to obesity and glucose metabolism. We aimed to assess whether the *MC4R* genotype affected longitudinal changes in body weight and glucose metabolism biomarkers among women with prior gestational diabetes mellitus (GDM). The *MC4R* genotype, postpartum weight reduction, and glycemic changes between after delivery and pregnancy were assessed in a cohort of 1208 Chinese women who had experienced GDM. The adiposity-increasing allele (C) of the *MC4R* variant rs6567160 was associated with greater postpartum increase of HbA1c (β = 0.08%; *P* = 0.03) and 2-hour OGTT glucose concentrations (β = 0.25 mmol/L; *P* = 0.02). In addition, we found an interaction between the *MC4R* genotype and postpartum weight reduction on changes in fasting plasma glucose (*P*-interaction = 0.03). We found that the *MC4R* genotype was associated with postpartum glycemic changes; and the association with fasting glucose were significantly modified by postpartum weight reduction in women who had experienced GDM.

## Introduction

In women, body weight changes considerably during pregnancy, usually increasing during the gestational period and decreasing at postpartum period^1^. In epidemiology studies, low postpartum weight reduction has been consistently related to abnormal glucose metabolism and an increased risk of type 2 diabetes in later life^2^. The positive relationship between postpartum weight reduction and risk of diabetes was found to be even stronger among women with a history of gestational diabetes mellitus (GDM)^3–6^.

The melanocortin-4 receptor (MC4R) is a G protein-coupled receptor that plays a pivotal role in regulating food intake, energy expenditure and adiposity, primarily through modulation of sympathetic outflow^7–13^. Rare mutations in the *MC4R* gene have been found to cause morbid obesity in humans^12^, and common polymorphisms were recently related to higher BMI in various populations in genome-wide association studies (GWAS)^14, 15^. In addition, the *MC4R* genotypes have been also related to increased risk of insulin resistance and type 2 diabetes^7, 14–17^. However, little is known whether the *MC4R* genotypes affect longitudinal changes in body weight and glucose metabolism during and after pregnancy among women.

In thus far one of the largest cohorts of women with a history of GDM, we examined the associations of an obesity-associated *MC4R* variant with postpartum changes in body weight and glucose metabolism. We particularly assessed the interaction between the *MC4R* genotype and postpartum weight reduction in relation to the changes of glucose metabolism.

## Results

The present study included a total of 1208 Chinese women with prior GDM. The frequency of the adiposity-increasing allele of *MC4R* rs6567160 (C allele) was 23%, and the genotype distribution fit the Hardy-Weinberg equilibrium (*P* = 0.26). The characteristics of participants during the pregnancy and at the postpartum survey by the *MC4R* genotype are presented in Table 1. The *MC4R* genotype was not related to measures of glucose metabolism (fasting glucose, 2-h OGTT glucose, and HbA1c) during pregnancy, but showed significant and positive associations (*P* < 0.05) with 2-h OGTT glucose, HbA1c, weight and BMI measured at postpartum survey.

**Table 1:** Characteristics of participants during pregnancy and at postpartum survey by *MC4R* rs6567160 genotype^1,2^.

Pre-pregnancy	*n* = 1208	TT (*n* = 713)	CT (*n* = 439)	CC (*n* = 56)	*P*-value^3^
Age, y	30.1 ± 3.5	30.1 ± 3.5	30.0 ± 3.5	30.4 ± 3.6	0.94
Pre-pregnancy weight^4^, kg	59.4 ± 9.1	58.9 ± 9.1	59.9 ± 9.1	61.1 ± 9.3	0.02
Pre-pregnancy BMI^4^, kg/m²	23.1 ± 3.3	23.0 ± 3.3	23.2 ± 3.3	23.4 ± 3.4	0.20
Characteristic of GDM screening^5^					
Fasting glucose, mmol/L	5.3 ± 0.81	5.3 ± 0.8	5.4 ± 0.8	5.4 ± 0.7	0.19
2-h OGTT glucose, mmol/L	9.2 ± 1.3	9.2 ± 1.3	9.1 ± 1.3	9.1 ± 1.2	0.34
HbA1c, %	5.8 ± 0.7	5.8 ± 0.7	5.9 ± 0.6	5.8 ± 0.6	0.12
Postpartum survey					
Number of follow-up, y	2.8 ± 0.9	2.8 ± 0.9	2.8 ± 0.9	2.8 ± 0.9	0.96
Age, y	32.4 ± 3.5	32.4 ± 3.5	32.3 ± 3.5	32.7 ± 3.6	0.96
Weight, kg	62.1 ± 10.8	61.4 ± 10.5	63.1 ± 11.0	64.4 ± 11.4	<0.01
BMI, kg/m²	24.2 ± 3.9	24.0 ± 3.9	24.4 ± 4.0	24.7 ± 4.3	0.04
Fasting glucose, mmol/L	5.4 ± 1.0	5.4 ± 0.9	5.4 ± 1.1	5.5 ± 0.8	0.18
2-h OGTT glucose, mmol/L	7.1 ± 2.5	7.0 ± 2.3	7.2 ± 2.7	7.8 ± 3.0	0.02
HbA1c, %	5.6 ± 0.8	5.6 ± 0.7	5.7 ± 0.8	5.8 ± 1.0	<0.01
Postpartum weight reduction (kg/y)^6^					
Tertile 1 (*n* = 402)	−2.8 ± 1.6	−2.8 ± 1.7	−2.8 ± 1.5	−3.1 ± 1.2	0.43
Tertile 2 (*n* = 403)	−6.3 ± 0.9	−6.3 ± 0.9	−6.5 ± 0.9	−6.4 ± 1.0	0.03
Tertile 3 (*n* = 403)	−11.5 ± 3.4	−11.6 ± 3.6	−11.6 ± 3.3	−10.9 ± 2.3	0.70

^1^Values are mean ± SD.

^2^CC, homozygote for risk allele; CT, heterozygote; HbA1c, glycated hemoglobin; *MC4R*, Melanocortin-4 receptor; OGTT, 2-h 75-g oral glucose tolerance test; TT, wild type.

^3^Linear regression models between characteristics of participants and *MC4R* genotype as a continuous variable [the dosage of the risk allele (0, 1 or 2), i.e 0 means no risk allele (TT), 1 means one risk allele (CT), and 2 means two risk alleles (CC)].

^4^Self-reported pre-pregnancy weight.

^5^Values are at initial screening test at 26–30 gestational weeks.

^6^Tertile 1 mean: −2.8 kg/y (min −4.8 kg; max: 4.8 kg); Tertile 2 mean: −6.4 kg/y (min −7.9 kg; max: −4.8 kg); Tertile 3 mean: −11.5 kg/y (min −29.6 kg; max: −7.9 kg).

### MC4R and glycemic changes

We further analyzed the associations of the *MC4R* genotype with reduction in body weight and measures of glucose metabolism from pregnancy to postpartum survey. Carriers of the adiposity-increasing allele (C) of rs6567160 showed less significant reduction of 2-h OGTT and HbA1c after adjustment for covariates including age at postpartum, pre-pregnancy BMI, follow-up years since delivery, concentrations of its respective biomarker (fasting or 2-hour plasma glucose or HbA1c) during pregnancy, number of children delivered, family history of diabetes (*P* = 0.03 for change of HbA1c and *P* = 0.02 for change of 2-h OGTT glucose) (Table 2). There was no significant association between the *MC4R* genotype and postpartum changes from the time point when women were diagnosed with GDM to postpartum 1–5 years in body weight and fasting glucose levels after adjustments.

**Table 2:** Association between *MC4R* genotype and postpartum changes in body weight and measures of glucose metabolism^1,2^.

	TT (*n* = 713)	CT (*n* = 439)	CC (*n* = 56)	Unadjusted *β*	Unadjusted *P*-value	Adjusted *β*	*P*-value^3^
Postpartum weight reduction^4^, kg/y	−7.0 ± 4.3	−6.8 ± 4.2	−6.5 ± 3.6	0.21	0.31	0.20	0.27
Change of BMI^5^, kg/m^2^	1.0 ± 2.3	1.2 ± 2.3	1.3 ± 2.5	0.20	0.08	0.20	0.13
Change of fasting glucose^5^, nmol/L	0.1 ± 1.0	0.1 ± 1.2	0.1 ± 0.9	0.01	0.82	0.03	0.46
Change of 2-h OGTT glucose^5^, mmol/L	−2.2 ± 2.3	−2.0 ± 2.6	−1.4 ± 2.9	0.34	<0.01	0.25	0.02
Change of HbA1c^5^, %	−0.2 ± 0.9	−0.2 ± 1.0	−0.0 ± 1.1	0.06	0.24	0.09	0.03

^1^Values are unadjusted mean ± SD.

^2^CC, homozygote for risk allele; CT, heterozygote; HbA1c, glycated hemoglobin; *MC4R*, Melanocortin-4 receptor; OGTT, 2-h 75-g oral glucose tolerance test; TT, wild type.

^3^Linear Regression. Models were adjusted for age at postpartum, pre-pregnancy BMI, follow-up years since delivery, level of the corresponding biomarkers during the pregnancy, number of children delivered, and family history of diabetes.

^4^Weight change from delivery to postpartum 1–5 y.

^5^Change from the time point when women were diagnosed with GDM to postpartum 1–5 y.

### Postpartum weight reduction and glycemic changes

**Figure 1:**
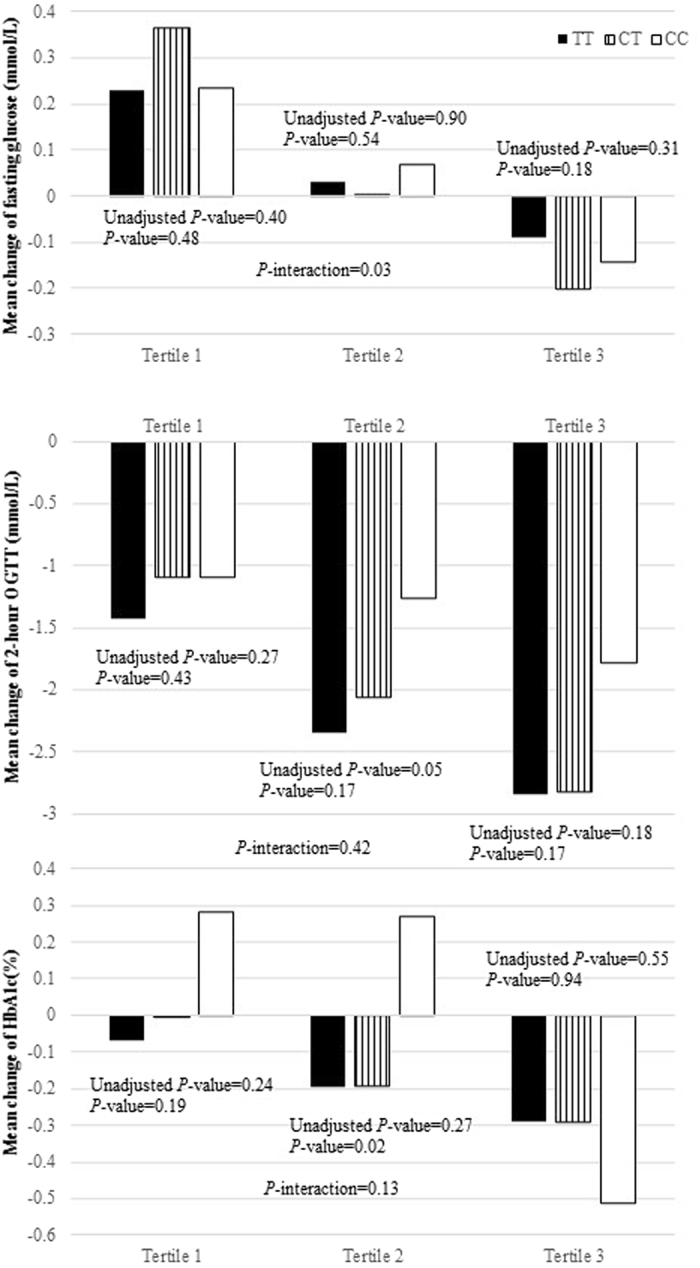
Mean change of glucose metabolism biomarkers by *MC4R* and tertiles of postpartum weight reduction. Values are mean change of fasting glucose (**a**), change of 2-h OGTT (**b**), change of HbA1c (**c**) ± SD. From the lowest to highest weight reduction; Tertile 1 mean: −2.8 kg/y (min −4.8 kg; max: 4.8 kg); Tertile 2 mean: −6.4 kg/y (min −7.9 kg; max: −4.8 kg); Tertile 3 mean: −11.5 kg/y (min −29.6 kg; max: −7.9 kg). Associations between levels of glucose biomarkers and *MC4R* by tertiles of postpartum weight reduction were analyzed by general linear regression models adjusted for age at postpartum, pre-pregnancy BMI, follow-up years since delivery, level of the corresponding biomarkers during the pregnancy, number of children delivered, and family history of diabetes. HbA1c, glycated hemoglobin; OGTT, 2-h 75-g oral glucose tolerance test.

In the study population, postpartum weight reduction showed significant associations with decreased levels of fasting glucose, 2-h OGTT, and HbA1c (all *P* values < 0.05) from pregnancy to postpartum survey. Adjustment for age at postpartum, pre-pregnancy BMI, follow-up years since delivery, level of the corresponding biomarkers during the pregnancy, number of children delivered, and family history of diabetes did not appreciably change the results. Women who were in the highest tertile of postpartum weight reduction presented −0.13 mmol/L, −2.79 mmol/L, and −0.30% changes in fasting glucose, 2-h OGTT, and HbA1c, respectively; while the corresponding changes were 0.28 mmol/L, −1.28 mmol/L, and −0.03%, respectively among women in the lowest tertile of postpartum weight reduction. The mean changes of glucose metabolism biomarkers stratified by *MC4R* genotype and postpartum weight reduction were shown in Fig. 1. In sensitivity analyses, we did not observe difference in longitudinal changes of these glucose metabolism markers according to the length of follow-up.

### Interaction analysis

We then tested the interaction between the *MC4R* genotype and postpartum weight reduction in relation to changes in biomarkers of glucose metabolism. Significant interactions were observed between *MC4R* SNP rs6567160 and postpartum weight reduction on changes of fasting glucose levels (*P*-interaction = 0.03). The adiposity-increasing allele (C) was related to increased fasting glucose concentrations among women in the lowest tertile of postpartum weight reduction; whereas an opposite-directional association was observed among women in the highest tertile (Fig. 1). The *MC4R* genotype did not interact with postpartum weight reduction on changes of 2-h OGTT and HbA1c levels.

## Discussion

In this study of a large cohort of Chinese women with a history of GDM, we found that the obesity-associated *MC4R* genotype was significantly related to greater reduction in HbA1c and 2-h OGTT levels during postpartum. In addition, we found that the *MC4R* genotype significantly interacted with postpartum weight reduction in relation to changes in fasting glucose levels.

Women usually gain around 20% body weight during pregnancy^1, 2^ and lose weight during postpartum period. Compelling evidence suggests that postpartum weight reduction plays a pivotal role in determining glucose metabolism after delivery and affects long-term risk of type 2 diabetes in later life, especially among those with a history of GDM^2–6, 18^. Consistent with previous studies^2^, in our cohort, a greater postpartum weight reduction was found to be related to significant decreases in fasting glucose, HbA1c and 2-h OGTT concentrations. Even though the *MC4R* genotype was significantly related to greater body weight, consistent with previous studies^7, 15^, it was not associated with postpartum weight reduction in our study.

The *MC4R* genotype was significantly related to changes in biomarkers of glucose metabolism including HbA1c and 2-h OGTT from pregnancy to postpartum survey, independent of pre-pregnancy BMI. Melanocortin-4 receptor, which is coded by the *MC4R* gene, is a key protein in regulation of energy balance, adiposity, and glucose metabolism^10, 16, 17^. The common variants in the *MC4R* gene have been previously related to measures of glucose metabolism such as insulin resistance^16^, insulin sensitivity^11^, and HbA1c^19^. Our data suggest that the *MC4R* genotype may also play a role in regulating postpartum change patterns of glycemic profiles caused by pregnancy.

Intriguingly, we found that the *MC4R* genotype was differently associated with changes in fasting glucose according to levels of postpartum weight reduction. The adiposity-predisposing allele (C) was associated with increased fasting glucose concentrations among women in the lowest tertile of postpartum weight reduction, but was related to decreased fasting glucose concentrations among women in the highest tertile. Such opposite genetic effects could be partly explained by the “differential susceptibility hypothesis”^20, 21^, which suggests that genes may be conceptualized as “plastic”, because genetic risk can be modified by environmental factors^20, 21^, such as change in body weight. We assume that the magnitude of postpartum weight reduction may differently affect expression or activity of the *MC4R* gene associated with the variant, and subsequently affect glucose metabolism during postpartum period.

To the best of our knowledge, the present study is among the first to show the effects of the *MC4R* genotype on postpartum changes in glucose metabolism among women with a history of GDM. The major strengths of our study include a large sample size, and longitudinally measured markers of glucose metabolism including fasting glucose, 2-h OGTT, and HbA1c at two time points (during and after pregnancy). We acknowledged some limitations of this study. For example, few lifestyle covariates were collected during the pregnancy and at the postpartum period; therefore, these potential confounders such as breast-feeding status and the duration of breast-feeding status could not be adjusted for in our analyses. In addition, we acknowledged that the participation rate was relatively low, however there were no differences between the women with GDM at 26–30 gestational weeks who returned and those who did not return, with regard to age, fasting glucose, 2-h glucose concentrations, and the prevalence of impaired glucose tolerance and diabetes. We excluded women with diagnosed diabetes, and such exclusion might affect the effect size of the associations. However, the number of women with diabetes was small (n = 15); therefore the influence would be moderate. We acknowledged that multiple outcomes were analyzed; however, these measures were correlated, and correction for multiple testing might increase type 2 error. We recognized that measurement errors might bias the associations, especially when the sample size is relatively small^22^, however in our study, we have a good sample size and the clinical phenotypes were objectively measured. Even though, scientific replications of our findings are essentially important. Our study was focused on a GWAS-identified gene, even though, we acknowledged that the ratio of false-positive to false-negative findings might be higher as compared with genome-wide analysis^23^. Therefore, the results from our study would be interpreted with caution and may not be generalizable to other populations and women without GDM.

In conclusion, our results for the first time indicate that the *MC4R* genotype is associated with postpartum changes in glucose metabolism among women with a history of GDM; and the genetic effects on glycemic changes might be modified by postpartum weight reduction.

## Methods

### Study population

The Tianjin Gestational Diabetes Mellitus Prevention Program is a retrospective cohort study in women with a history of GDM at 1–5 y after delivery. Detailed information of the study has been described elsewhere^24–27^. Briefly, all pregnant women in the urban areas of Tianjin, China, who had diagnosed with GDM at 26–30 gestational weeks (according to World Health Organization criteria)^28^ between 2005 and 2009 (*n* = 4,644) were invited to participate in a postpartum survey from August 2009 to July 2011^25^. A total of 3381 women were excluded due particularly to the impossibility of being contacted, to have refused, and to not meet study criteria^24^. The exclusion criteria were: age <20 or ≥50 y; had a diagnosis of diabetes (2-h 75-g oral glucose tolerance test (OGTT) glucose concentration ≥11.1 mmol/L or fasting glucose ≥7.0 mmol/L); presence of any chronic diseases that could reduce the life expectancy or the ability to participate in the study, such as cancer and cardiovascular diseases; taking medicines that alter 2-h OGTT glucose; pregnant during the follow-up period; and unable to give informed consent. Therefore 1,263 GDM women completed the postpartum survey (participation rate 27%). We excluded 55 women without genotype information due to lack of DNA samples and these women were not different in clinical characteristics and biochemical markers from those included in the final analysis. Thus, a total of 1,208 participants were analyzed in the present study. A previous study showed there were no differences in 2-h OGTT glucose concentration, fasting glucose concentration, and the prevalence of impaired glucose tolerance and diabetes at 26–30 gestational weeks between women who returned postpartum survey and those who did not^27^. In the postpartum survey, each eligible participant had a physical examination and answered a self-administered questionnaire^24^. The questionnaire contained information about participants’ pregnancy outcomes (pre-pregnancy weight, gestational weight gain, and number of children delivered), medical records of GDM history, family history of diseases (diabetes, hypertension, stroke, and cancer), medical history (pregnancy hypertension, diabetes, hypertension and hypercholesterolemia), and socio-demographic information^25^. The study protocols were guided by the Ethical Principles and Guidelines for the Protection of Human Subjects of Research and the study was approved by the Human Subjects Committee of the Tianjin Women’s and Children’s Health Center. A informed consent was obtained from all participants^24^.

### Assessment of anthropometrics

In the postpartum survey, body weight and height were measured using the standardized protocol. Pre-pregnancy and current BMI were calculated by dividing pre-pregnancy or current weight (in kilograms) by the square of height (in meters).

The postpartum weight retention (weight change between pre-pregnancy and 1–5 y postpartum) was divided in two parts: the gestational weight gain and postpartum weight reduction. For the present study, we used only the postpartum weight reduction divided by the number of follow-up years since delivery.

### Assessment of biomarkers of glucose metabolism and covariates

The biomarker concentrations were measured in plasma both during the pregnancy (26–30 gestational weeks, the time point when the women were diagnosed with GDM) and at the postpartum survey. Blood samples were collected from all participants after an overnight fast of at least 12 hours. Fasting glucose and 2-h 75 g OGTT glucose were measured using an automatic analyzer (TBA-120FR; Toshiba, Japan). Glycated hemoglobin (HbA1c) was measured using Automatic Glycohaemoglobin Analyzer (ADAMS A1c HA-8160; Arkray, Japan). Changes in biomarkers were calculated as the difference in biomarker concentrations between postpartum 1–5 y (at postpartum survey) and pregnancy.

### DNA extraction, SNP selection, and genotyping

Genomic DNA was extracted from the buffy coat fraction of centrifuged blood using a QIAamp Blood Maxi Kit (Qiagen, Chatsworth, CA). *MC4R* single nucleotide polymorphism (SNP) rs6567160 was determined by quantitative real-time TaqMan polymerase chain reaction (Applied Biosystems, Foster City, CA). The success rate of genotyping was over 98%. For quality control, 10% of the samples were re-genotyped with more than 99% concordance.

### Statistical analysis

The Hardy-Weinberg equilibrium of the genotype was examined by a χ^2^ test (*P* > 0.05). The normal distribution of the variables was evaluated using the Kolmogorov-Smirnov test. Data were expressed as the mean and standard deviation (SD). The associations of the *MC4R* genotype and postpartum weight reduction with changes in fasting glucose, 2-h OGTT glucose, and HbA1c were analyzed using general linear regression models adjusted for age at postpartum, pre-pregnancy BMI, follow-up years since delivery, number of children delivered, respective biomarker concentrations measured when women were diagnosed with GDM at 26–30 gestational weeks, and family history of diabetes. The *MC4R* genotype was analyzed as a continuous variable (additive model), which indicates that the associations between *MC4R* genotype and glucose metabolism biomarkers are increased γ-fold for genotype *T/C* and by 2γ-fold for genotype *C/C*. The interaction between the *MC4R* genotype and postpartum weight reduction was tested by introducing a product term for these variables in the models. We also analyzed the associations between the *MC4R* genotypes and glucose metabolism biomarkers by the tertiles of postpartum weight reduction (from the lowest to the highest: Tertile 1, −4.8 to 4.8; Tertile 2, −7.9 to −4.8; and Tertile 3, −29.6 to −7.9 kg/y), using linear regression model, adjusted for age at postpartum, pre-pregnancy BMI, follow-up years, level of the corresponding biomarkers during the pregnancy, number of children delivered, and family history of diabetes. In addition, sensitivity analyses were performed to check whether the number of follow-up years could influence the relationship between changes in biomarker concentrations and postpartum weight reduction. All statistical analyses were performed using SAS statistical software (version 9.4; SAS Institute, Inc., Cary, NC, USA). *P* < 0.05 was considered statistically significant.
